# Quantification of angiogenesis in estrogen receptor-positive and negative breast carcinoma

**DOI:** 10.1186/1477-7800-4-22

**Published:** 2007-08-24

**Authors:** JB Parentes-Vieira, PV Lopes-Costa, CG Pires, AR dos Santos, JD Pereira-Filho, BB da Silva

**Affiliations:** 1 Department of Gynecology, Federal University of Piauí, Teresina, Piauí, Brazil; 2 Avenida Elias João Tajra, 1260, Apt. 600, Bairro Jockey Club, CEP: 64049-300, Teresina, Piauí, Brazil

## Abstract

**Background:**

The objective of this study was to evaluate angiogenesis according to CD34 antigen expression in estrogen receptor (ER)-positive and negative breast carcinomas.

**Methods:**

This study comprised 64 cases of infiltrating ductal carcinoma in postmenopausal women divided into two groups: Group A: ER-positive, n = 35; and Group B: ER-negative, n = 29. The anti-CD34 monoclonal antibody was used as a marker for endothelial cells. Microvessel count was carried out in 10 fields per slide using a 40× objective lens (magnification 400×). Statistical analysis of the data was performed using Student's t-test (p < 0.05).

**Results:**

The mean number of vessels stained with the anti-CD34 antibody in the estrogen receptor-positive and negative tumors was 23.51 ± 1.15 and 40.24 ± 0.42, respectively. The number of microvessels was significantly greater in the estrogen receptor-negative tumors (p < 0.001).

**Conclusion:**

ER-negative tumors have significantly greater CD34 antigen expression compared to ER-positive tumors.

## Background

There has been growing interest in the important role played by the estrogen receptor in the clinical care of patients with breast cancer [1]. Only around 6 – 10% of normal breast epithelial cells express estrogen receptors (ER), while around 60% of primary breast cancers are ER-positive [2,3]. This possibility makes the definition of estrogen receptor status a routine procedure in the management of a patient with breast cancer, primarily as a predictive and then as a prognostic factor [4]. A relatively better prognosis may be expected in patients whose tumors express estrogen receptors compared with tumors that do not [5,6].

Estrogen, by interacting with ER, plays an important role not only in the regulation and differentiation of the normal breast epithelium but also in breast tumorigenesis [7]. Estrogen deprivation has been shown to result in a marked reduction in angiogenesis, which returns to pretreatment levels following estrogen replacement [8,9].

There is considerable experimental evidence showing that tumor growth is dependent on angiogenesis; moreover, a tumor will not grow to more than 1–2 mm, around 10^6 ^cells, nor metastasize if neovascularization from preexisting capillaries fails to occur [10,11].

In general, malignant tumors with poor prognosis were found to have high microvessel density (MVD) [12]. Several papers have shown that the quantification of angiogenesis by counting blood vessels provides an independent assessment of prognosis [13]. A correlation between ER status and microvessel count (MVC) in a breast tumor would be of great interest, useful not only for defining prognosis but also for the selection of patients with initial breast cancer for hormone therapy [7]. Some authors have reported a reduction in the expression of vascular endothelial growth factor (VEGF) in ER-positive breast tumors, principally in those with high levels of ER-alpha [14,15]. Other investigators have found no difference and others even same an increase in the MVC of ER-positive compared to ER-negative tumors [16,17]. Therefore, in view of these controversies, the present study evaluated angiogenesis and ER status in biopsy samples from pretreatment breast carcinomas.

## Methods

This study included tumor samples from 64 patients, who had been postmenopausal for at least two years and who were receiving care at the Mastology Department of the Federal University of Piauí. These patients were submitted to surgical treatment between 2004 and 2006 for estrogen receptor-positive and negative infiltrating ductal breast carcinoma. None of these patients had undergone any prior treatment. The study was approved by the Institutional Review Board of the Federal University of Piauí and the patients signed an informed consent form prior to undergoing diagnostic biopsy. The samples were obtained from incisional biopsy carried out prior to definitive treatment. The biopsy samples were fixed in buffered formalin and stained with hematoxylin-eosin for confirmation of the diagnosis of invasive ductal carcinoma. All samples were then submitted to immunohistochemical analysis to evaluate estrogen receptor status. Tumors with nuclear staining that was semiquantitatively classified as high (>10% immunoreactive cells) were considered positive [18].

The cases were then divided into two groups: Group A: ER-positive, n = 35; and Group B: ER-negative, n = 29. Patients ranged in age from 47 to 82 years (mean 52.40 years) in Group A and from 46 to 88 years (mean 58.38 years) in Group B. The size of the tumors in the two groups ranged from 2.5 to 5 cm, stage II, mean tumor size 3.36 and 3.26 in groups A and B, respectively. The groups were considered homogeneous with respect to age and to the size and histological grade of the tumor (Table 1). For CD34 antigen immunostaining, specific primary monoclonal antibody for CD34 was used (Anti-human Hematopoietic Progenitor Cell, CD34 Class II, Clone QBEnd 10, Code M 7165, DAKO Corporation, Carpinteria, CA, USA) at a dilution of 1:25 with bovine serum albumin (BSA). Antigen retrieval was carried out in a steamer containing sodium citrate buffer (pH = 6) at 90°C for 30 minutes. Sections were then incubated overnight with the primary specific antibody at 4°C. Color was developed using DAB (3-3' diaminobenzidine, SIGMA code 5637), a chromogenic substrate. Liver hemangiomas were used to test positive and negative controls for the immunohistochemical reaction.

**Table 1:** Characteristics of the patients in the study groups

**Age (years)**	Group A	Group B
Mean	52.40	58.38
SE	2.32	2.26
SD	13.75	12.18
P = 0.070: Student's t-test; P = 0.204: Mann-Whitney's non-parametric test		
		
**Tumor size (cm)**		
Mean	3.36	3.26
SE	0.21	0.22
SD	1.25	1.19
P = 0.746: Student's t-test; P = 0.833: Mann-Whitney's non-parametric test		
		
**Histological grade**		
	n (%)	n (%)
1	12 (34.29)	8 (27.59)
2	16 (45.71)	11 (37.93)
3	7 (20.00)	10 (34.48)
total	35 (100.00)	29 (100.00)
P = 0.426: Chi-square test		

Group A: ER-positive, n = 35; Group B: ER-negative, n = 29

SD: standard derivation; SE: standard error

Microvessel count was performed by two independent observers, who were blinded with respect to the patients' identities. A Nikon Eclipse E400 light microscope was used to count microvessels and was coupled to a color video camera that transmitted the image to a microcomputer and hence to a monitor, counting being performed with the aid of an image analysis software program (Imagelab). Initially, the areas with greater microvessel density were identified at a magnification of 40×. Microvessel count was performed in 10 fields in each of these areas of highest density (40× objective lens, 10× ocular lens), total magnification 400×. Units of vessel counts were identified according to the criteria established by Weidner et al [19], who described them as a group of brown-stained endothelial cells, clearly separated from the adjacent microvessels, tumoral cells and other conjunctive tissues. The vessel lumen, although usually present, was not a criterion used to define a microvessel, and red blood cells were not used to define vessel lumens. Partially identified vessels that were not completely contained within the fields under analysis were not considered in the vessel count. In each case, microvessel density consisted of the mean number of vessels counted in ten fields.

Student's t-test and the Mann-Whitney non-parametric test were used to establish homogeneity between the two groups with respect to age and tumor volume. The Chi-square test was used to evaluate the difference between the two groups with respect to histological grade (Table 1). Student's t-test was used to compare the mean number of vessels in the two groups (p < 0.05).

## Results

**Figure 1:**
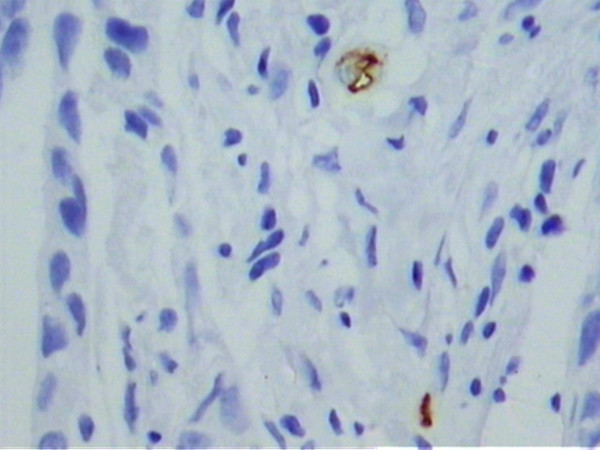
Microphotograph of a histological section of estrogen receptor-positive breast carcinoma (patient #5), showing sparse vessels stained with anti-CD34 (Original magnification, 400×).

**Figure 2:**
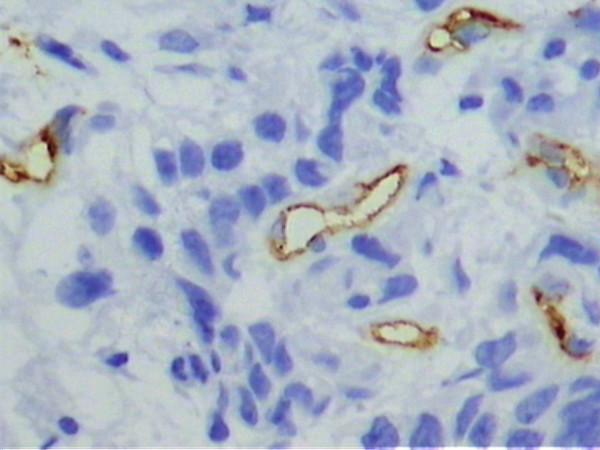
Microphotograph of a histological section of estrogen receptor-negative breast carcinoma (patient #8), showing a high concentration of microvessels stained with anti-CD34 (Original magnification, 400×).

Under light microscopy, varying degrees of vascular neoformation were found in the two groups. There were greater concentrations of microvessels stained brown by the anti-CD34 antibody in the tumor samples from the estrogen receptor-negative group compared to the estrogen receptor-positive tumors (Figures 1 and 2). Quantitative analyses of the microvessels in the 10 fields revealed a mean vessel count of 23.51 ± 0.72 in the estrogen receptor-positive tumors and 40.24 ± 1.54 in the estrogen receptor-negative tumors (Table 2). This difference was statistically significant (p < 0.001). There was excellent agreement and low interobserver variance between the two observers for the 64 specimens (R^2 ^= 0.99).

**Table 2:** Mean number of microvessels in estrogen receptor-positive (A) and estrogen receptor-negative (B) breast carcinoma.

Group	n	Mean	SE	SD	Maximum	Minimum
A	35	23.51	0.72	4.31	34.00	14.00
B	29	40.24*	1.54	8.31	61.00	21.00

* The difference was statistically significant (p < 0.001).

## Discussion

The growth and proliferation of tumor cells, as well as their metastatic dissemination, have been shown to be preceded and facilitated by the formation of new blood vessels from preexisting capillaries [11]. Angiogenesis has been considered an independent prognostic factor [8]; therefore, its assessment may provide additional information on the biological profile of the tumor, and may have applications in prognostic evaluation and as a therapeutic target in human breast carcinoma [13,20]. Nevertheless, although a higher density of microvessels is generally found in malignant breast tumors with the worst prognosis and estrogen receptor-negative tumors have a relatively poorer prognosis, conflicting reports have been published on the correlation between tumoral angiogenesis and ER status [5-7,12,15,16].

In the present study, microvessel count in estrogen receptor-negative breast carcinomas of postmenopausal women was significantly greater compared to estrogen receptor-positive breast carcinomas. The women in the two groups were homogenous with respect to age and to the size and histological grade of the tumor, which made comparison between the two groups feasible. The tumor specimens in the present study were obtained from wedge biopsy; therefore, it is unlikely that the results obtained simply reflect tumor heterogeneity, which is a recognized methodological problem [17]. Studies carried out to evaluate the effect of tumor heterogeneity in microvessel count in breast cancer specimens have focused on the use of core biopsies [17], which contain less tumor volume than the preoperative wedge biopsies used in this study. CD34 was selected from the available markers because it is a sensitive marker that stains the neoplastic endothelium more strongly than the normal endothelium [21].

The presence of a significantly lower number of vessels in the ER-positive breast tumors observed in the present study indicates a correlation between tumor angiogenesis and estrogen receptor status. Moreover, in an *in vivo *study using nude female mice, Ali et al. [15] showed that high levels of ER-alpha downregulate angiogenic factors VEGF and integrin alphavbeta3 (αvβ3), leading to inhibition of tumor angiogenesis.

On the other hand, Erdem et al. [16] failed to show any difference between mean MVD values in estrogen receptor-positive and negative tumors. The samples in that study consisted of archival specimens collected around 7–10 years previously. However, for some investigators, material kept in storage for a long time may result in loss of antigenicity for some markers [22]. Likewise, in samples obtained by core needle biopsy from breast tumors of 158 patients, Vamesu [7] showed that a high microvessel density was significantly more common in patients with ER-positive/PR-negative tumors. Nevertheless, the possible effect of tumor heterogeneity on microvessel counts in specimens originating from core biopsies should be emphasized [17] as well as the overexpression of variants of the subtypes of estrogen receptor that affect the regulation of tumor angiogenesis [23].

Of the multitude of growth factors that regulate angiogenesis, VEGF is believed to be the most important, whereas exon-deleted variants of ER-alpha, such as ERDelta3, a variant frequently overexpressed in breast cancer, may exert an undesirable effect, contributing significantly to VEGF production and thus exacerbating tumor growth *in vivo *[23]. Therefore, a better understanding of the correlation between VEGF and the subtypes of estrogen receptors and their variants in breast cancer, in combination with their prognostic importance, may lead to the development of therapeutic strategies directed against VEGF or its receptor.
